# Type 2 diabetes and cancer: A retrospective longitudinal comparative cohort study of disease sequence and comorbidity profiles

**DOI:** 10.1371/journal.pone.0350276

**Published:** 2026-06-10

**Authors:** Maha Alriyami, Amal Saki Malehi, Asil Al Kamyani, Fatema Al Jabri, Maha Alhosni, Khalid Al Saadi, Salim Al-Maqbali, Tahani K. Al-Shuaili, Abdullah Al-Futaisi

**Affiliations:** 1 Department of Biochemistry, College of Medicine and Health Sciences, Sultan Qaboos University, Muscat, Oman; 2 Department of Family Medicine and Public Health, College of Medicine and Health Sciences, Sultan Qaboos University, Muscat, Oman; 3 College of Medicine and Health Sciences, Sultan Qaboos University, Muscat, Oman; 4 Department of Dermatology, Oman Medical Specialty Board, Muscat, Oman; 5 Department of Accident and Emergency Medicine, Medical City for Military and Security Services (MCMSS), Muscat, Oman; 6 Department of Family Medicine, Medical City for Military and Security Services (MCMSS), Muscat, Oman; 7 Department of Medicine, College of Medicine and Health Sciences, Sultan Qaboos University, Muscat, Oman; Athens Medical Group, Psychiko Clinic, GREECE

## Abstract

Type 2 Diabetes Mellitus (T2DM) and cancer are major non-communicable diseases that impose a substantial dual burden when they co-occur. However, this dual burden remains understudied and the majority of existing studies investigate T2DM as a comorbidity without distinguishing whether it is pre-existing at the time of cancer diagnosis or newly-onset following diagnosis. This study aimed to investigate the co-occurrence of T2DM and cancer and to assess differences in laboratory variables, comorbidities, complications and overall survival according to the sequence of diagnosis. This retrospective cohort study with longitudinal follow-up included patients diagnosed with both T2DM and cancer. A total of 213 adult patients who meet the inclusion criteria were categorized based on the sequence of diagnosis: T2DM prior to cancer (n = 131) or cancer prior to T2DM (n = 82). The prevalence of T2DM among in-patients with cancer was 27.4%. Statistical analyses were performed to compare clinical and laboratory characteristics between groups. Breast cancer and colon cancers were the most common cancer types. Most patients were diagnosed with T2DM prior to cancer onset and were significantly older than those diagnosed with cancer first (*p* = 0.02). Male patients had a higher risk of mortality compared to females (HR = 1.96, *p* = 0.03). Among patients diagnosed with cancer prior to T2DM, males had significantly higher diastolic blood pressure (*p* = 0.02), while females had significantly higher total cholesterol levels (*p* = 0.04). No significant differences were observed in comorbidities or complications between groups (all *p >* 0.05). There were no significant differences in overall survival between the two diagnostic-order groups (*p* = 0.03). Moreover, separate survival analysis for breast, colon, lung and gastric cancers showed no significant differences between patients diagnosed with T2DM before cancer and those diagnosed with cancer before T2DM (all *p >* 0.05). Future studies are warranted to confirm these findings and to better understand the burden of the co-occurrences of T2DM and cancer at national level.

## Introduction

Cancer and Type 2 diabetes mellitus (T2DM) are two non-communicable diseases (NCDs) that have been targets of the 2030 Sustainable Development Goals (SDGs), particularly SDG 3.4, which aims to reduce premature mortality from NCDs by one-third by 2030 [1]. Cancer is the leading cause of premature mortality worldwide [2]. Although advances in early detection and treatment have improved survival for many cancer sub-types, it continues to impose a substantial public health burden, demanding sustained and effective control strategies [3]. Importantly, cancer outcomes are influenced by coexisting chronic conditions, particularly metabolic diseases such as Type 2 diabetes mellitus (T2DM), which can affect disease progression, treatment response, and survival.

Diabetes mellitus (DM) is also a growing global public health challenge. The total number of affected adults globally has been projected to increase to 643 million by 2030 affecting individuals, with most affected individuals residing in in low- and middle-income countries [4]. Moreover, DM accounted for 6.7 million deaths in 2021 [4]. T2DM accounts for more than 90% of DM cases worldwide [5,6]. The continued increase in the overall number of T2DM cases can be partially attributed to shifts in societal lifestyles, as well as to enhanced diagnostic strategies and improved survival rates [7].

T2DM and cancer frequently co-occur [8]. The association between the two diseases has been attributed to their shared genetic, metabolic, and lifestyle risk factors including age, gender, low levels of physical activity, obesity, alcohol consumption, and smoking [8]. These factors predispose individuals to an increased risk of developing both T2DM and various types of cancer. Moreover, the coexistence of the two diseases result in a compounded health burden and poses significant challenges for the management of both conditions. For example, sustaining optimal glycemic control is challenging in patients with T2DM undergoing cancer-related interventions, including surgery, glucocorticoid therapy, and treatment-related symptoms such as nausea and vomiting [9]. Conversely, cancer treatments have been associated with poorer outcomes in patients with T2DM [10–12]. Furthermore, T2DM has been associated with substantially increased mortality among patients with cancer [12,13]. This highlights the need to prioritize prevention and early detection of both diseases, particularly in high-risk populations.

Oman, like many countries in the Gulf region, is undergoing rapid socioeconomic development and lifestyle changes that are contributing to a rising burden of NCDs. However, the dual burden of T2DM and cancer remains understudied in the region. Most existing studies investigate T2DM as a comorbidity without distinguishing whether it is pre-existing at the time of cancer diagnosis or newly diagnosed thereafter, despite the clinical relevance of this distinction. The primary aim of this study is to shift the analytical focus from co-prevalence to disease trajectories which may provide additional clinically relevant insights that might not be revealed when T2DM is considered only as a comorbidity.

The objectives of the study are to: ***1.*** Investigate the prevalence of T2DM among cancer patients in a cohort from Oman. ***2.*** To compare the laboratory parameters, comorbidities, and complications among cancer patients stratified by the diagnostic sequence of the two diseases (T2DM diagnosed prior to cancer vs T2DM diagnosed after cancer). ***3.*** To compare the overall survival across the two groups.

We hypothesize that patients diagnosed with T2DM prior to cancer may differ in laboratory parameters, comorbidity burden, and survival from those who develop T2DM after a cancer diagnosis. These differences may reflect the effects of chronic metabolic dysfunction preceding cancer in the former group, whereas, cancer and its treatment may contribute to metabolic disturbances in the latter. Understanding the potential variations associated with the order of diagnosis may reveal important epidemiological patterns and disease trajectories which may help physicians anticipate complications and inform more tailored intervention strategies. Moreover, it may also inform policymakers in planning and screening strategies and guidelines for specific cancer types in high-risk populations.

## Methods

### Ethics approval

Ethical approval was obtained from the local Institutional Review Board (IRB): the Medical Research Ethics Committee (MREC) of the College of Medicine and Health Sciences, Sultan Qaboos University (REF. No. 2530) on July 15, 2021. Every effort was made to adhere to the ethical guidelines of the Declaration of Helsinki, developed by the World Medical Association.

### Study design and setting

This study is an institution-based, retrospective cohort study with longitudinal follow-up conducted by collecting data from the medical records of all patients admitted to the oncology unit during the time-period from January 1st, 2018, to December 31st, 2019, at Sultan Qaboos University Hospital (SQUH), Oman. The study period was chosen to avoid potential confounding related to COVID-19 pandemic-associated disruptions in healthcare delivery, patient management, and public health measures. Medical records were accessed retrospectively between September 2021 to April 2022 to extract clinical, laboratory, and comorbidity data and were re-accessed between March 2025 and June 2025 to update and complete survival outcome data through extended longitudinal follow-up. SQUH is a tertiary care and teaching hospital in Oman, established in 1990, and offers a comprehensive range of services, including primary, secondary, and tertiary care. SQUH receives referrals from different parts of the country.

### Sample size

The minimum required sample size was calculated using a 95% confidence level and a margin of error of 5%. Assuming an expected prevalence of 20% based on published epidemiological data on T2DM in Oman [14], the required sample size was 246 patients. As described below the total number of patients who fulfilled the inclusion criteria was 213; therefore, all eligible patients were included in the analysis.

### Data collection

Data were collected retrospectively from the electronic medical records of patients treated at the oncology unit. All patients aged ≥ 12 years admitted to the oncology unit during the study period (January 1, 2018, to December 31, 2019) with a cancer diagnosis, were considered for inclusion (n = 985). Cancer diagnoses were extracted from hospital medical records and were mapped to International Classification of Diseases, 10th Revision (ICD-10), Chapter II (malignant neoplasms) codes (C00–C97). Coding was based on the documented primary anatomic site of the tumor. Where the anatomic site or sub-type was not specified in the medical records, unspecified ICD-10 codes (e.g., CXX.9) were assigned. The level of diagnostic specificity reflects the detail available in the records.

Among these patients with cancer, those with a diagnosis of T2DM were eligible for inclusion. Patients with confirmed DM were initially identified based on records, and pregnant females, patients with Type 1 diabetes mellitus (T1DM), and those with gestational DM were excluded. The T2DM diagnosis was also subsequently confirmed by review of clinical documentation and laboratory data, in accordance with the American Diabetes Association (ADA) diagnostic criteria routinely followed at the Sultan Qaboos University Hospital. These criteria included a fasting blood glucose (FBG) level ≥ 7.0 mmol/L (126 mg/dL) after ≥ 8 hours of fasting, a random blood glucose (RBG) level > 11.1 mmol/L (200 mg/dL), or an HbA1c level > 6.5%. Moreover, for further confirmation, T2DM diagnosis was also confirmed by checking patients’ medications for antidiabetic drugs.

**Fig 1:**
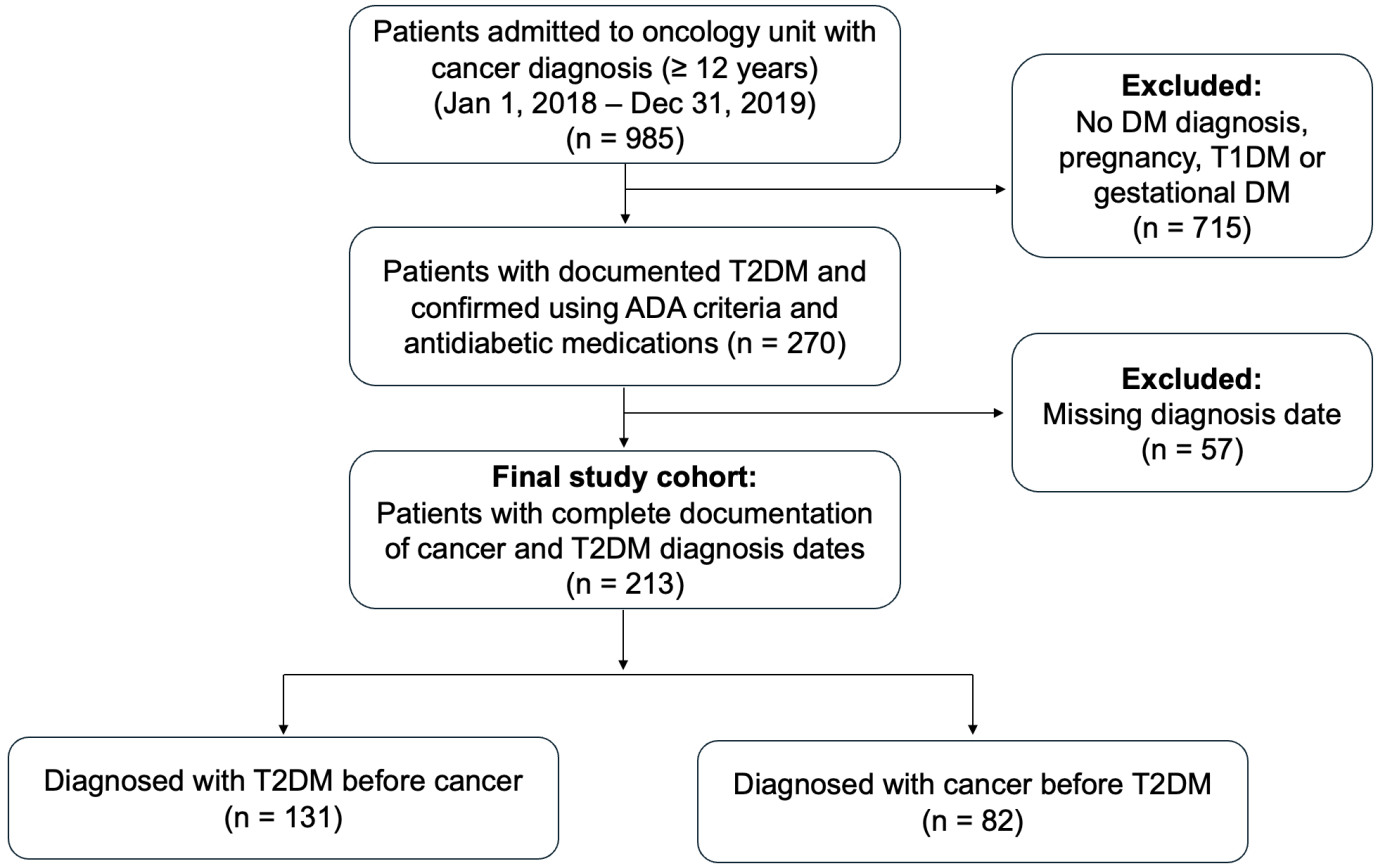
Flow diagram of cohort selection from hospital records, including inclusion and exclusion criteria.

A total of 270 eligible adult patients with cancer and T2DM were initially identified. Patients with missing documentation of the date of diabetes or cancer diagnosis (n = 57) were excluded resulting in a final study cohort of n = 213 patients. The final study cohort was then categorized based on the order of diagnosis into two groups: Diagnosed with T2DM before cancer (n = 131) and diagnosed with cancer before T2DM (n = 82) (Fig 1). Demographic characteristics, cancer type, laboratory results, diabetes-related complications, comorbidities, and medication use were recorded from the medical records (S1 Table).

### Statistical analysis

Categorical variables were reported by frequency (percentage), and quantitative variables were provided by mean ± SD. Kolmogorov-Smirnov’s normality test was performed on quantitative variables. Prevalence estimates were calculated as proportions with 95% confidence intervals. To compare the categorical variables between two groups categorized based on the order of diagnosis (T2DM prior to cancer group vs. cancer prior to T2DM group), Chi-square test was employed. Independent samples *t*-tests and Mann-Whitney *t*ests were used to compare quantitative variables between two groups. Multiple binary logistic regression was conducted to investigate the relationship between comorbidities & complications and the order of diabetes diagnosis with adjustment for age, sex, and cancer type.

Survival time was calculated from the diagnostic date of cancer (as time zero) to either death (event) or last follow-up visit (right censor), measured in months. The Kaplan-Meier method was used to estimate the survival function, and the Log-rank test was implemented to compare the survival curves of the two groups based on their order of diabetes diagnosis. Furthermore, the Cox regression model was performed by adjusting for significant variables based on the findings of the univariate analysis.

The *p* value **<**0.05 was regarded as statistically significant for other tests. All statistical analysis was performed using SPSS version 29 (IBM Corp., Armonk, NY, USA).

## Results

### Demographics

We reviewed the medical records of all adult patients with cancer who were admitted to the oncology unit during a two-year study period from January 1, 2018, to December 31, 2019. During the study period, a total of 985 adult patients were admitted to the oncology unit with a cancer diagnosis. Among these, 270 patients had T2DM, yielding an inpatient prevalence of 27.4% (95% CI: 24.6%− 30.2%). After excluding patients with missing dates of diagnosis, 213 patients were included in the final analysis and were categorized based on the order of diagnosis: T2DM prior to cancer (n = 131) and cancer prior to T2DM (n = 82) (Fig 1).

The mean age of patients diagnosed with both cancer and T2DM was 61.91 ± 12.31 years (range: 32–91 years). Of these, 42.7% were males (n = 91) and 57.3% were females (n = 122). The mean age for males was 64.04 ± 11.92 years and for females was 60.31 ± 12.39 years (Table 1). Overall, 15 patients (7.0%) were current smokers, and 3 patients (1.4%) were current alcohol consumers (Table 1). The mean body mass index (BMI) was 28.96 ± 11.7 kg/m^2^, corresponding to the overweight category. The mean BMI among males was 25.52 ± 6.51 kg/m^2^ and the mean BMI among females was 31.53 ± 13.09 kg/m^2^ (Table 1).

**Table 1:** Demographic of the study cohort affected by type 2 diabetes mellitus and cancer (2018- 2019) (n = 213).

Demographic and Clinical Variables		N (%) n = 213	Mean ± SD
Age			61.91 ± 12.31
Gender	Male	91 (42.7)	
	Female	122 (57.3)	
Smoking		15 (6.9)	
Alcohol consumption		3 (1.4)	
Body mass index (BMI)			28.96 ± 11.17

### Clinical characteristics by order of diagnosis

Patients diagnosed with T2DM prior to cancer were significantly older than those diagnosed with cancer first (64.89 ± 11.29 vs. 57.14 ± 12.43 years, respectively; *p* = 0.02). Sex was not significantly associated with the order of diagnosis of T2DM and cancer (*p* = 0.06) (Table 2). Smoking status and alcohol consumption were also not significantly associated with diagnostic order (*p* = 0.53 and *p* = 0.94, respectively). Similarly, BMI was not associated with whether T2DM or cancer was diagnosed first (*p* = 0.74) (Table 2). Male patients diagnosed with cancer prior to T2DM had significantly higher diastolic blood pressure (DBP) compared to those diagnosed with T2DM first (81.46 ± 25.49 vs. 69.17 ± 15.05, respectively; *p* = 0.02). No significant difference in DBP was observed among females. Systolic blood pressure (SBP) did not differ significantly between diagnostic-order groups in either males or females (all *p* > 0.05).

**Table 2:** Comparison of demographic and clinical characteristics between patients with T2DM and cancer categorized based on the order of diagnosis (n = 213).

Demographic and Clinical Variables		Diagnosed first with T2DM before cancer		*p*-value*	Adjusted *p*-value (BH)^$^
		Yes (N = 131) N (%)	No (N = 82) N (%)		
Gender	Male	65 (49.6)	26 (31.7)	**0.010**	0.06
	Female	66 (50.4)	56 (68.3)		
Smoking		11 (20.4)	4 (11.8)	0.30	0.53
Alcohol consumption		2 (3.6)	1 (3.1)	0.90	0.94
		**Mean ± SD**	**Mean ± SD**	***p*-value**	
Age		**64.89 ± 11.29**	**57.14 ± 12.43**	**< 0.001** ^**¶**^	**0.02**
BMI		29.22 ± 13.01	28.96 ± 7.44	0.61^¥^	0.74
Systolic blood pressure (SBP)	Male	127.17 ± 29.75	130.31 ± 24.51	0.64^¶^ 0.52^¶^	0.74
	Female	137.58 ± 24.05	134.66 ± 26.88		0.70
Diastolic blood pressure (DBP)	Male	**69.17 ± 15.05**	**81.46 ± 25.49**	**0.003** ^**¶**^	**0.02**
	Female	70.92 ± 13.0	76.18 ± 16.04	0.02^¶^	0.07
Fasting blood glucose (mmol/L)	Male	9.02 ± 3.23	9.87 ± 2.99	0.33^¶^	0.54
	Female	9.62 ± 2.04	11.02 ± 3.45	0.01^¶^	0.03
Random blood glucose level (mmol/L)	Male	10.47 ± 4.51	9.82 ± 3.94	0.70^¥^	0.77
	Female	11.02 ± 3.44	9.62 ± 3.45	0.02^¥^	0.06
Glycated hemoglobin (HbA1c)	Male	7.07 ± 1.33	7.26 ± 1.95	0.60^¥^	0.77
	Female	**8.11 ± 1.84**	**6.93 ± 2.00**	**< 0.001** ^**¥**^	**0.01**
Total cholesterol (TC) (mmol/L)	Male	4.47 ± 1.28	5.13 ± 1.50	0.11^¶^	0.25
	Female	**4.71 ± 1.15**	**5.52 ± 1.60**	**0.01** ^**¶**^	**0.04**
HDL (mmol/L)	Male	1.05 ± 0.31	1.18 ± 0.32	0.15^¶^	0.29
	Female	1.15 ± 0.39	1.22 ± 0.33	0.46^¶^	0.71
LDL (mmol/L)	Male	2.66 ± 1.19	3.22 ± 1.35	0.13^¶^	0.27
	Female	2.72 ± 0.88	3.24 ± 1.00	0.02^¶^	0.05
Triglycerides (TG) (mmol/L)	Male	1.68 ± 0.86	1.61 ± 0.99	0.52^¥^	0.75
	Female	2.21 ± 2.92	1.83 ± 1.10	0.91^¥^	0.91

* Chi-square test.

^¶^ Independent samples t-test.

^¥^ Mann-Whitney test.

^$^ Benjamini-Hochberg procedure.

Fasting blood glucose (FBG) was significantly higher among females diagnosed with cancer prior to T2DM compared to patients diagnosed with T2DM first (11.02 ± 3.45 vs. 9.62 ± 2.04, respectively; *p* = 0.03). Whereas glycated hemoglobin (HbA1c) was significantly higher among females diagnosed with T2DM prior to cancer (8.11 ± 1.84 vs. 6.93 ± 2.00, respectively; *p* = 0.01). Moreover, mean total cholesterol (TC) was significantly higher among females diagnosed with cancer first (5.52 ± 1.60 vs. 4.71 ± 1.15 respectively; *p* = 0.04). Moreover, it was within the borderline-high range. No other parameter showed significant differences after BH adjustment (all *p* > 0.05) (Table 2).

### Types of cancer by order of diagnosis

The study cohort was affected by a spectrum of cancer types. The most common types were breast cancer (C50.9) (n = 83, 39.0%), colon cancer (C18.9) (n = 47, 22.1%), lung (C34.9) (n = 19, 8.9%) and gastric cancer (C16.9) (n = 15, 7.0%) (Table 3). Among females (n = 122), breast cancer was the most common malignancy (n = 81, 66.4%), followed by colon cancer (n = 20, 16.4%). In contrast, among males, colon cancer was the most common type (n = 27, 29.7%), followed by lung cancer (n = 16, 17.6%) (S2 Table).

**Table 3:** Documented types of cancer for the study cohort diagnosed with both T2DM and cancer and categorized by order of diagnosis (n = 213). Cancer types are classified according to ICD-10 malignant neoplasm codes.

ICD-10 block	Cancer diagnoses documented in hospital records (ICD-10)	Frequency (N = 213) N (%)	Diagnosed first with T2DM before cancer	
			Yes (N = 131) N (%)	No (N = 82) N (%)
Breast (C50)	Breast cancer, unspecified (C50.9)	83 (38.9)	47 (35.9)	36 (43.9)
Digestive organs (C15-C26)	Gastric cancer, unspecified (C16.9)	15 (7.0)	9 (6.9)	6 (7.3)
	Duodenal cancer (C17.0)	2 (0.9)	2 (1.5)	0 (0.0)
	Jejunum cancer (C17.1)	1 (0.5%)	0 (0.0)	1 (1.2)
	Colon, unspecified (C18.9)	47 (22.1)	31 (23.7)	16 (19.5)
	Gallbladder cancer (C23)	1 (0.5)	1 (0.8)	0 (0.0)
	Hepatocellular carcinoma (C22.0)	3 (1.4)	3 (2.3)	0 (0.0)
	Biliary tract cancer, unspecified (C24.9)	3 (1.4)	3 (2.3)	0 (0.0)
Pancreas (C25)	Pancreatic cancer, unspecified (C25.9)	4 (1.9)	3 (2.3)	1 (1.2)
Respiratory organs (C33–C34)	Lung cancer, unspecified (C34.9)	19 (8.9)	11 (8.4)	8 (9.8)
Larynx (C32)	Laryngeal cancer, unspecified (C32.9)	1 (0.5)	1 (0.8)	0 (0.0)
Lip, oral cavity and pharynx (C00–C14)	Nasopharyngeal cancer, unspecified (C11.9)	2 (0.9)	1 (0.8)	1 (1.2)
	Retromolar cancer (C06.2)	1 (0.5)	0 (0.0)	1 (1.2)
Brain (C71)	Brain cancer, unspecified (C71.9)	2 (0.9)	1 (0.8)	1 (1.2)
	Cerebellar astrocytoma (C71.6)	1 (0.5)	1 (0.8)	0 (0.0)
Thyroid (C73)	Thyroid cancer (C73)	3 (1.4)	1 (0.8)	2 (2.4)
Other endocrine glands (C75)	Parathyroid cancer (C75.0)	1 (0.5)	0 (0.0)	1 (1.2)
Urinary organs (C64-C68)	Bladder cancer, unspecified (C67.9)	8 (3.8)	4 (3.1)	4 (4.9)
Prostate (C61)	Prostate cancer (C61)	7 (3.3)	5 (3.8)	2 (2.4)
Ovary (C56)	Ovarian cancer (C56)	1 (0.5)	0 (0.0)	1 (1.2)
Lymphoid/hematopoietic (C81-C96)	Lymphoma, unspecified (C85.9)	6 (2.8)	5 (3.8)	1 (1.2)
Skin (C43)	Melanoma, unspecified (C43.9)	1 (0.5)	1 (0.8)	0 (0.0)
Retroperitoneum and peritoneum (C48)	Retroperitoneal sarcoma (C48.0)	1 (0.5)	0 (0.0)	1 (1.2)
Neuroendocrine tumors (ICD-10-CM*: C7A)	Neuroendocrine tumor, unspecified (C7A.00)	1 (0.5)	1 (0.8)	0 (0.0)

*ICD-10, Clinical modification.

Investigation of cancer types in the two diagnostic order groups (T2DM diagnosed first vs. cancer diagnosed first) showed that breast cancer was the predominant malignancy in both groups (n = 47, 35.9% and n = 36, 43.9%, respectively), followed by colon cancer (n = 31, 23.7% and n = 16, 19.5%, respectively), lung cancer (n = 11, 8.4% and n = 8, 9.8%, respectively) and gastric cancer (n = 9, 6.9% and n = 6, 7.3%, respectively).

### Comorbidities and complications by order of diagnosis

The study cohort experienced several comorbidities and complications. The most common was cardiovascular diseases (CVD) affecting 55.9% of patients (n = 119), followed by hypoglycemia, which occurred in 21.6% of patients (n = 46). The least frequent complication was diabetic foot ulcer, observed in one patient only (0.5%) (Table 4).

**Table 4:** Comparison of comorbidities and complications between patients categorized according to the order of diagnosis (n = 213).

Comorbidities and Complications (ICD-10)	Frequency (N = 213) N (%)	Diagnosed first with diabetes before cancer		*p*-value^*^	*Adjusted p*-value^**^ Binary logistic
		Yes (N = 131) N (%)	No (N = 82) N (%)		
Hypoglycemia (E11.649)	46 (21.6)	29 (27.1)	17 (21.0)	0.33	0.21
Diabetic ketoacidosis (DKA) (E11.10)	2 (0.93)	2 (2.5)	0 (0.0)	0.18	0.99
Hyperglycemic hyperosmolar state (HHS) (E11.00)	4 (1.9)	3 (2.9)	1 (1.2)	0.45	0.58
Microvascular diabetic complications^$^ (E11.2-E11.5)	41 (19.2)	27 (23.7)	14 (17.3)	0.28	0.78
Diabetic foot ulcer (E11.621)	1 (0.5)	1 (0.9)	0 (0.0)	0.90	0.39
Cardiovascular diseases (I20-I25, I50)	119 (55.9)	83 (77.6)	36 (60.0)	0.02	0.23
Chronic kidney diseases CKD (N18.x)	20 (9.4)	10 (17.5)	10 (25.6)	0.34	0.62
Chronic liver diseases CLD (K70-K77)	12 (5.6)	10 (16.9)	2 (5.9)	0.13	0.22
Chronic gastrointestinal diseases (K90–K93)	18 (8.5)	12 (21.4)	6 (16.7)	0.57	0.83
Stroke and cerebrovascular diseases (I60-I69)^&^	28 (13.1)	15 (26.3)	13 (30.2)	0.67	0.59
Pulmonary disease (J40–J47)	26 (12.2)	16 (26.2)	10 (24.4)	0.83	0.69

^$^ Microvascular diabetes complication: Diabetic nephropathy (E11.21), diabetic retinopathy (E11.3x) and diabetic neuropathy (E11.40/E11.42).

^&^ Stroke and cerebrovascular diseases: Ischemic Stroke (I63), Intracerebral Hemorrhage (I61), Cerebral infarction sequelae (I69).

** Multiple binary logistic regression with adjustment for age, sex, and cancer type.

* Chi-square test.

Patients diagnosed with T2DM prior to cancer had a higher prevalence of most comorbidities and complications than those diagnosed with cancer first. However, after adjustment using binary logistic regression, none of the examined comorbidities were significantly associated with the order of diagnosis (*p* > 0.05) (Table 4).

### Survival time by order of diagnosis

**Fig 2:**
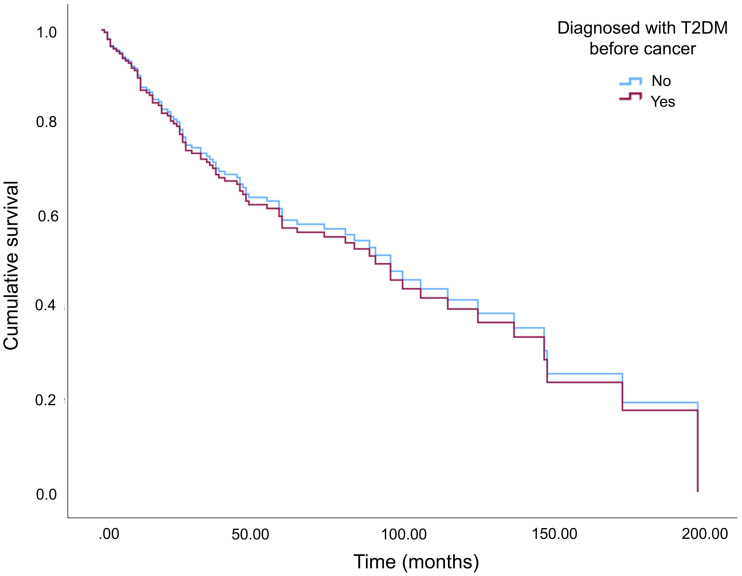
Kaplan-Meier Survival Curves based on order of diagnosis of the study cohort (n = 213).

**Fig 3:**
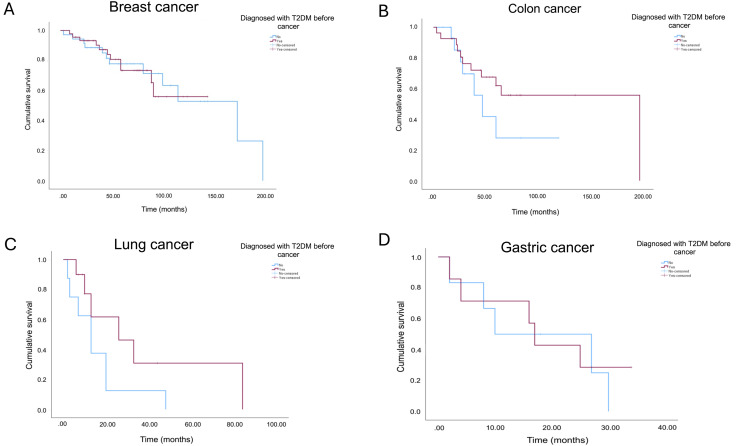
Kaplan-Meier Survival Curves based on order of diagnosis of the study cohort (n = 213) for A. Breast cancer, B. Colon cancer, C. Lung cancer, D. Gastric cancers as the three big groups of cancer in the study cohort.

A Kaplan-Meier survival analysis was performed to estimate survival probabilities over time for the two diagnostic order groups. The mean survival time for patients diagnosed with T2DM prior to cancer was 76.55 months (mean 95% CI: 65.03 to 88.08). In comparison, patients diagnosed with cancer first had a mean survival time of 84.39 months (95% CI: 70.55–88.85). The log-rank test showed no significant difference in survival between the two groups (*p* = 0.33) (Table 5, Fig 2). Comparing the survival time for patients diagnosed with T2DM and the most prevalent types of cancers in the study cohort (breast, colon, gastric and lung cancers) showed no significant difference in survival between the two diagnostic order groups (*p* = 0.93, 0.27, 0.76, 0.11, respectively) (Table 5, Fig 3).

**Table 5:** Comparison of survival time among the study cohort based on the order of diagnosis (n = 213).

Order of diagnosis		Mean survival time (months) 95% CI	Median survival time (months) 95% CI	Log rank test statistic	*p*- value
T2DM prior to overall cancer	Yes	76.55 (65.03,88.08)	74 (59.37, 88.63)	0.94	0.33
	No	84.39 (70.55, 88.85)	72 (53.24, 90.77)		
T2DM prior to breast cancer	Yes	106.79 (88.79,124.78)	–	0.01	0.93
	No	129.89 (99.62, 160.15)	173.0 (96.24, 249.76)		
T2DM prior to colon cancer	Yes	125.74 (89.63, 161.84)	198.0 (-)	1.22	0.27
	No	60.71 (35.10, 86.31)	47.0 (27.80, 66.20)		
T2DM prior to gastric cancer	Yes	18.86 (9.95, 27.77)	17.0 (14.43, 19.57)	0.09	0.76
	No	17.58 (7.52, 27.65)	10.0 (0.0, 28.24)		
T2DM prior to lung cancer	Yes	38.91 (13.49, 64.34)	26.0 (1.88, 50.12)	2.50	0.11
	No	15.75 (5.54, 25.96)	13.0 (4.95, 21.05)		

**Fig 4:**
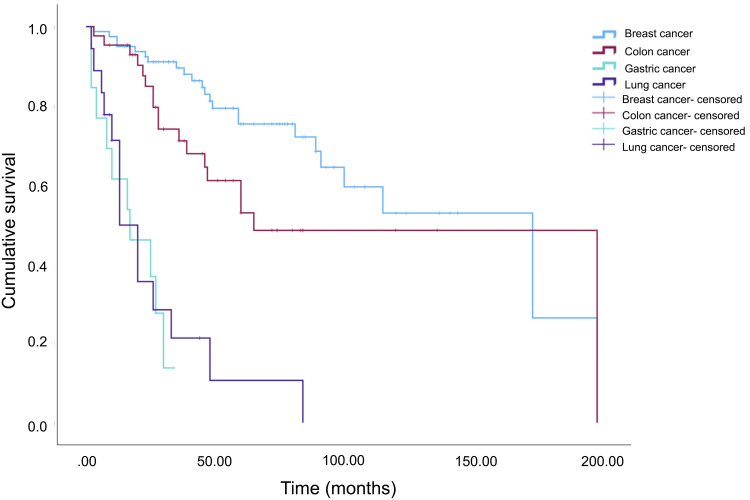
Plot of Kaplan-Meier product limit estimates of breast, colon, and gastric and lung cancers as the four most prevalent types of cancer in the study cohort (log-rank test = 77.41, *p*-value < 0.001).

A Kaplan-Meier survival analysis also demonstrated a significant difference in survival across the most prevalent types of cancers in the cohort. Lung and gastric cancers exhibited significantly poorer survival outcomes compared to breast and colon cancers (log-rank test = 77.41, *p*-value < 0.001) (Fig 4). The observed variations in survival may reflect differences in stage at diagnosis and tumor biology as lung and gastric cancers usually present at more advanced stages compared to breast cancer.

Plot of Kaplan-Meier product limit estimates of patients who were first diagnosed with diabetes and those who were diagnosed with diabetes after cancer in the study population (log-rank test = 0.94, *p*-value = 0.33). Patients who were lost to follow-up or did not experience the event by the end of the study period are represented by censoring indicated on the curves by tick marks.

A Cox proportional hazards regression model was performed adjusting for variables that were significant in the univariate analysis. The results showed that patients diagnosed with T2DM prior to cancer had a 1.22-fold higher risk of death than patients diagnosed with cancer first. However, this was not significantly different (HR = 1.22, *p* = 0.55). A significant difference was observed between males and females (HR = 1.96, *p* = 0.03), with males having 1.96 times higher risk of death than females. None of the other investigated variables showed a significant association with the risk of death (Table 6).

**Table 6:** Cox proportional hazards regression analysis examining the association between clinical variables and overall survival in the study cohort (n = 213).

Variables	Subgroups	coefficient	Standard error (SE)	Hazard ratio (HR)	95.0% CI for HR		*p*-value
					Lower	Upper	
Diagnosed with T2DM prior to cancer	Yes	0.20	0.34	1.22	0.63	2.36	0.55
	No*	---	---	---	---	---	
Sex	Male	0.67	0.30	1.96	1.09	3.55	0.03
	Female*	---	---	---	---	---	
Age		−0.01	0.01	0.99	0.97	1.01	0.36
Systolic blood pressure (SBP)		−0.02	0.01	0.98	0.96	0.99	0.01
Diastolic blood pressure (DBP)		0.00	0.02	1.00	0.97	1.04	0.83
Glycated hemoglobin (HbA1c)		−0.14	0.10	0.87	0.72	1.05	0.15
Total cholesterol (TC)		−0.23	0.10	0.97	0.79	1.19	0.78

*Reference categories, SE standard error; HR hazard ratio.

## Discussion

In this study, we investigated the clinical characteristics and survival outcomes of patients with co-occurring T2DM and cancer in a cohort from Oman. We reviewed the medical records of patients admitted to an oncology unit and were diagnosed with both T2DM and cancer between January 1, 2018, and December 31, 2019. The prevalence of T2DM among inpatients with cancer was 27.4%. A total of 213 patients with complete diagnosis dates were included and categorized by diagnostic order (T2DM first vs. cancer first). Patients in the T2DM-first group were significantly older; sex was not associated with diagnostic order, and the predominant malignancies in both groups were breast, colon, lung, and gastric cancers. Regarding clinical characteristics, male in the cancer-first group had significantly higher DBP than those in the T2DM-first group. Among females, FBG was significantly higher in the cancer-first group, whereas HbA1c was significantly higher in the T2DM- first group; TC was also significantly higher in females in the cancer-first group. Comorbidities were not associated with the diagnostic order. Survival analysis for patients with T2DM and the most prevalent cancers (breast, colon, gastric and lung cancers) showed no significant difference between groups.

Although the study period may not fully reflect recent changes in health trends and clinical practice, the findings remain relevant because the examined relationships and variations by order of diagnosis of T2DM and cancer are based on fundamental disease mechanisms that are unlikely to change substantially over time. In addition, these results provide a reference point for comparisons with the pre-COVID-19 pandemic period. The oncology unit where patients were admitted is part of the Internal Medicine Department at Sultan Qaboos University Hospital and it consists of four oncologists. This unit receives referrals from across the country and manages patients diagnosed within the hospital.

The prevalence of T2DM among inpatient cancer patients during the study period was relatively high (27.4%), indicating that slightly more than one-quarter of patients had T2DM. This prevalence is higher than the global prevalence of 8–18% (varying by cancer site) reported in meta-analyses [11,15]. The relatively high prevalence highlights the overlap and shared comorbidity between T2DM and cancer. Moreover, it may also reflect the high prevalence of T2DM in Oman, which is projected to increase from 15.2% in 2020 to up to 18% by 2030 and 23.8% by 2050 [16].

After excluding patients with missing diagnosis dates, 213 patients were included in the final analysis. The mean age of the cohort was 61.91 ± 12.31 years, and the majority were females (57.3%). Patients diagnosed with T2DM prior to cancer were significantly older than those diagnosed with cancer first (*p* < 0.02). Sex was not significantly associated with the order of diagnosis of T2DM and cancer (*p* = 0.06). This is different from what was previously reported in a Dutch cohort study, which suggested that T2DM is more likely to precede cancer diagnosis in males. In contrast, previous studies have reported that females may exhibit an increased risk of cancer several years after a diagnosis of DM [17]. There is growing evidence that T2DM-related metabolic disturbances, such as hyperglycemia, hyperinsulinemia, and inflammation, are associated with cancer etiology [8,18]. T2DM may promote cancer growth and metastasis by impairing immune function [6,19]. Moreover, hyperglycemia may contribute to cancer development by inducing DNA damage through increased oxidative stress and inflammation [6]. T2DM has also been associated with a higher risk of breast, liver, pancreatic, and bladder cancers [20].

Breast, colon, lung and gastric cancers were the most common malignancies observed in the cohort (38.9%, 22.1%, 8.9% and 7% respectively). These types of cancers were also the most frequent in both diagnostic-order groups. Patients with T2DM diagnosed prior to cancer had a higher prevalence of several comorbidities and complications, including hypoglycemia, microvascular diabetic complications, cardiovascular diseases, and chronic gastrointestinal diseases. However, these conditions were not significantly different.

Among females, breast cancer (66.4%) was the predominant malignancy, followed by colon cancer (16.4%) (S1 Table). These findings are consistent with previous reports indicating that breast, colorectal, and lung cancers are among the most frequent diagnosed cancer overall and among female patients [21]. In males, the most common cancer type in the study cohort was colon cancer (29.7%), followed by lung cancer (17.6%). This pattern is also consistent with previous reports showing that colorectal and lung cancers are the second and third most common cancers among males, after prostate cancer [21]. Globally, breast cancer is the predominant cancer type among women (11.6% of all cancers worldwide) [2], and Oman is no exception as in 2022 breast cancer accounted for approximately 34.9% of all cancer cases among Omani women [22,23]. Moreover, colorectal cancer is also one of the most prevalent cancers globally (9.6% of all cancers) [2]. In Oman, the three most common cancer types among both males and females, based on the registered cases in 2022, were breast cancer, colorectal cancer, and non-Hodgkin lymphoma [24]. This could explain the statistical appearance of these two types of cancer, regardless of the patient’s diabetes status.

In terms of clinical parameters, diastolic blood pressure was elevated and exceeded the diagnostic threshold for hypertension. Moreover, it was significantly higher in males diagnosed with cancer prior to diabetes (*p* = 0.02), suggesting increased peripheral vascular resistance in this group. One possible explanation is cancer-associated allostatic load (AL), defined as the cumulative physiological burden resulting from chronic stress and systemic inflammation [25]. Cancer progression and treatment may therefore elevate diastolic blood pressure. In addition, steroid-containing chemotherapies have been associated with the development of hypertension in cancer patients [26]. Contrariwise, individuals diagnosed with T2DM prior to cancer are more likely to undergo regular blood pressure monitoring and early antihypertensive management, which may contribute to better blood pressure control in this group.

In terms of diabetes-related parameters, females diagnosed with T2DM prior to cancer exhibited significantly higher HbA_1_c levels, indicating poorer long-term glycemic control. This finding likely reflects the chronic nature of T2DM; however, it may also be influenced by the shift in clinical priority following cancer diagnosis, which may contribute to metabolic stress and worsen glycemic control in this group. Previous studies have linked elevated HbA_1_c levels after cancer diagnosis and treatment to a lack of integrated care, competing care priorities, and poorer self-management by the patients themselves who are managing more than one chronic condition [27,28]. On the other hand, females diagnosed with cancer first exhibited significantly higher fasting blood glucose (*p* = 0.03). It has been previously reported that non-DM female patients undergoing breast cancer treatment show a significant rise in FBG, suggesting acute treatment-related hyperglycemia [29].The observed pattern of glycemic parameters suggests different metabolic profile based on the order of diagnosis, with cancer first females exhibiting more acute disturbance, while those with pre-existing T2DM demonstrate poorer long-term control. Further studies are required to investigate and confirm these associations.

In terms of lipid profile, significantly higher TC (*p* = 0.04) and borderline LDL (*p* = 0.05) levels were observed in female patients diagnosed with cancer prior to T2DM. These differences are clinically relevant as the TC levels exceeded clinical thresholds, while LDL levels approached ranges associated with increased cardiovascular risk. These data suggest a more atherogenic lipid profile in females diagnosed with cancer first, which may reflect cancer-associated metabolic alterations. Possible mechanisms include systemic inflammation and the effect of cancer therapies [30]. In contrast, patients diagnosed with T2DM first may benefit from ongoing metabolic management, including lipid-lowering therapies such as statins, which may contribute to better lipid control and mitigate the cancer-associated dyslipidemia. Further investigations are required to confirm these findings and to explore possible mechanisms.

Furthermore, the sex-specific differences in glycemic control, and TC are unlikely to be due to T2DM treatment alone as all patients in the cohort were on statin and antihypertensive drugs. However, it is more likely that these differences are more likely influenced by the type of cancer and its treatment. In this cohort, as the predominant type of cancer among female patients was breast cancer (66.4%, S2 Table). Breast cancer and its associated treatments have been reported to impair glucose metabolism primarily through increased insulin resistance and in some cases reduced insulin secretion, contributing to hyperglycemia [31]. Moreover, breast cancer has been previously shown to affect lipid metabolism profoundly. Furthermore, genes related to lipid metabolism were found to be upregulated in breast tissues prior to cancer diagnosis [32]. Nevertheless, these sex-related trends warrant further research.

Both DM and cancer contribute to morbidity and premature mortality [13]. T2DM has been associated with higher risk of mortality, particularly in breast cancer patients [13]. Survival analysis on the study cohort showed no significant difference in overall survival between the two diagnostic-order groups (*p* = 0.33). Similarly, survival analysis for patients with T2DM and the most common cancer types (breast, colon, gastric and lung cancers) also showed no significant differences between diagnostic order-groups. These finding suggest that the sequence of diagnosis does not independently influence survival outcomes. This could be due to the small sample size of the study cohort that might have limited the statistical power to detect survival differences. Moreover, the lack of significant differences in comorbidities and complications between the two diagnostic-order groups may have decreased potential differences in survival outcomes.

The Cox proportional hazards model showed that male sex was associated with a higher risk of mortality compared with females (HR = 1.96). Moreover, patients diagnosed with T2DM prior to cancer had a higher estimated risk of death compared with those diagnosed with cancer first; however, this association was not statistically significant (HR = 1.22, *p* = 0.55). This lack of statistical significance maybe related to the small sample size.

DM, particularly T2DM, has been associated with an increased risk of several cancers [33,34]. Symptoms include hyperinsulinemia, chronic inflammation, and hyperglycemia caused by DM, has been also associated with malignancies [35,36]. These associations have promoted interest in cancer screening programs among patients with T2DM, in which age- sex-appropriate cancer screening was recommended to be part of routine diabetes care [37]. However, it should be noted that observational evidence alone cannot determine the causal impact or effectiveness of such screening strategies. Moreover, some evidence indicates that patients with DM may participate in screening programs less than those without DM. For instance, a study from the Friuli Venezia Giulia region of Italy reported lower participation in colorectal cancer screening among individuals with DM despite higher adenoma detection rates [38]. In Oman, national cancer screening programs primarily target breast and colorectal cancer, using defined eligibility criteria to promote early detection [39,40]. Since T2DM has a high prevalence in Oman, encouraging cancer screening among T2DM patients may facilitate early detection and improved outcomes.

## Limitations

The retrospective nature of this study entails several limitations that should be considered when interpreting the findings. ***1.*** The analysis was based on data from a single oncology unit collected from a two-year period (January 1, 2018, to December 31, 2019); therefore, the results may not be fully generalizable to other healthcare settings, populations, or time periods. Nevertheless, the findings may serve as a reference point for comparison with data from the pre-COVID-19 pandemic period. ***2.*** The relatively small sample size may limit statistical power and reduce the precision of the observed associations. ***3.*** Reliance on existing patient records introduces the potential for information bias, due to errors, inconsistencies, or missing key information within clinical documentation. ***4.*** Important co-founders (such as obesity, cancer treatment regimes, DM management strategies, and socioeconomic factors) were not fully captured in the medical records and could not be accounted for in the analysis. ***5.*** Misclassification bias is possible due to the use of recorded diagnosis dates, which may not accurately reflect the true onset of the disease. Both conditions may have been present but undiagnosed for extended periods prior to formal documentation, potentially affecting the inferred order of diagnosis. ***6.*** Survival bias should be considered, as the study cohort may represent a selected group of patients who survived long enough to develop and be diagnosed with the second disease. These limitations highlight the need for larger, multicenter, and prospective longitudinal studies to more comprehensively evaluate the co-occurrence of T2DM and cancer, clarify their temporal sequence and assess associated clinical outcomes.

## Conclusion

This study highlights the clinical burden of coexisting T2DM and cancer, demonstrating that the sequence of diagnosis might be associated with differences in patients’ characteristics and metabolic profiles. These findings point to the need for more integrated and management approaches that address both oncologic and metabolic demands simultaneously. Enhanced coordination between specialties and improved patients support are essential to optimize glycemic control in this population.

Further large, multicenter prospective studies are necessary to better understand the co-occurrence of the two diseases and to evaluate strategies for improving long-term outcomes. Such research could enable care-providers to anticipate potential complications and to tailor treatment strategies accordingly. Moreover, such studies may reveal novel epidemiological insights and disease trajectories that may inform public health in planning, including screening practices and guidelines for high-risk populations.

### Declaration of generative AI and AI-assisted technologies in the writing process

During the preparation of this work, the author(s) used ChatGPT in order to check grammar, spelling, and improved sentence flow. After using this tool, the author(s) reviewed and edited the content as needed and take(s) full responsibility for the content of the publication.

## Supporting information

S1 Table Study Data Set. (DOCX)

S2 Table Cancer types of the study cohort. Types of cancer among patients diagnosed with both T2DM and cancer, categorized by gender and order of diagnosis (females: n = 122 and males: n = 91).
(XLSX)
