# Supplementary material for: Type 2 diabetes and cancer: A retrospective longitudinal comparative cohort study of disease sequence and comorbidity profiles
Source: PLoS One. 2026 Jun 10;21(6):e0350276. doi: 10.1371/journal.pone.0350276 (PMC13252760; doi:10.1371/journal.pone.0350276)
Supplement: S1 Table — (DOCX) [file pone.0350276.s001.docx]

Supplementary Material

Supplementary Table S. 1. Types of cancer among patients diagnosed with both T2DM and cancer, categorized by gender and order of diagnosis (females: n=122 and males: n=91).

| Gender | Cancer types | Diagnosed first with diabetes and then developed cancer | | Total  (N=122) |
| --- | --- | --- | --- | --- |
|  |  | Yes  N=62  (N%) | No  N=56  (N%) |  |
| Female | **Breast cancer** | **47 (71.2)** | **34 (60.7)** | **81 (66.4)** |
|  | Bile duct cancer | 0 (0) | 0 (0) | 0 (0) |
|  | Bladder cancer | 0 (0) | 1 (1.8) | 1 (0.8) |
|  | Brain cancer | 0 (0) | 1 (1.8) | 1 (0.8) |
|  | **Gastric cancer** | 1 (1.5) | **6 (10.7)** | 7 (5.7) |
|  | duodenal cancer | 2 (3.0) | 0 (0) | 2 (1.6) |
|  | Jejunal cancer | 0 (0) | 0 (0) | 0 (0) |
|  | Laryngeal cancer | 0 (0) | 0 (0) | 0 (0) |
|  | Lung cancer | 1 (1.5) | 2 (3.6) | 3 (2.5) |
|  | Ovarian cancer | 0 (0) | 1 (1.8) | 1 (0.8) |
|  | Pancreatic cancer | 0 (0) | 0 (0) | 0 (0) |
|  | Parathyroid cancer | 0 (0) | 1 (1.8) | 1 (0.8) |
|  | Prostate cancer | 0 (0) | 0 (0) | 0 (0) |
|  | Retro molar tooth Squamous cell carcinoma | 0 (0) | 0 (0) | 0 (0) |
|  | Retroperitoneal Sarcoma | 0 (0) | 1 (1.8) | 1 (0.8) |
|  | Thyroid cancer | 0 (0) | 2 (3.6) | 2 (1.6) |
|  | Colon cancer | **14 (21.2)** | **6 (10.7)** | **20 (16.4)** |
|  | Hepatocellular carcinoma | 0 (0) | 0 (0) | 0 (0) |
|  | Lymphoma | 0 (0) | 1 (1.8) | 1 (0.8) |
|  | Melanoma | 0 (0) | 0 (0) | 0 (0) |
|  | Cerebellar astrocytoma | 0 (0) | 0 (0) | 0 (0) |
|  | Neuroendocrine tumor | 0 (0) | 0 (0) | 0 (0) |
|  | Nasopharyngeal cancer | 0 (0) | 0 (0) | 0 (0) |
|  | Gall bladder cancer | 1 (1.5) | 0 (0) | 1 (0.8) |
| Gender | Cancer types | Diagnosed first with diabetes and then developed cancer | | Total  (N=91) |
|  |  | Yes  N=65  (N%) | No  N=26  (N%) |  |
| Male | Breast cancer | 0 (0) | 2 (7.7) | 2 (2.2) |
|  | Bile duct cancer | 3 (4.6) | 0 (0) | 3 (3.3) |
|  | Bladder cancer | 4 (6.2) | 3 (11.5) | 7 (7.7) |
|  | Brain cancer | 1 (1.5) | 0 (0) | 1 (1.1) |
|  | Gastric cancer | 8 (12.3) | 0 (0) | 8 (8.8) |
|  | Duodenal cancer | 0 (0) | 0 (0) | 0 (0) |
|  | Jejunal cancer | 0 (0) | 0 (0) | 0 (0) |
|  | Laryngeal cancer | 1 (1.5) | 0 (0) | 1 (1.1) |
|  | **Lung cancer** | **10 (15.4)** | **6 (23.1)** | **16 (17.6)** |
|  | Ovarian cancer | 0 (0) | 0 (0) | 0 (0) |
|  | Pancreatic cancer | 3 (4.6) | 1 (3.8) | 4 (4.4) |
|  | Parathyroid cancer | 0 (0) | 0 (0) | 0 (0) |
|  | Prostate cancer | 5 (7.7) | 2 (7.7) | 7 (7.7) |
|  | Retro molar tooth Squamous cell carcinoma | 0 (0) | 1 (3.8) | 1(1.1) |
|  | Retroperitoneal Sarcoma | 0 (0) | 0 (0) | 0 (0) |
|  | Thyroid cancer | 1 (1.5) | 0 (0) | 1(1.1) |
|  | Colon cancer | **17 (26.2)** | **10 (38.5)** | **27 (29.7)** |
|  | Hepatocellular carcinoma | 3 (4.6) | 0 (0) | 3 (3.3) |
|  | Lymphoma | 5 (7.7) | 0 (0) | 5 (5.5) |
|  | Melanoma | 1(1.5) | 0 (0) | 1 (1.1) |
|  | Cerebellar astrocytoma | 1(1.5) | 0 (0) | 1 (1.1) |
|  | Neuroendocrine tumor | 1(1.5) | 0 (0) | 1 (1.1) |
|  | Nasopharyngeal cancer | 1(1.5) | 1 (3.8) | 2 (2.2) |
|  | Gall bladder cancer | 0 (0) | 0 (0) | 0 (0) |
